# Women’s experiences of the assessment and management of urinary tract infections during the COVID-19 pandemic: a qualitative analysis of free-text comments from a national survey in England

**DOI:** 10.1093/jacamr/dlaf241

**Published:** 2025-12-19

**Authors:** A’Mar Dababneh, Leigh N Sanyaolu, Haroon Ahmed, Dushyanthi Alagiyawanna, Donna M Lecky, Emily Cooper

**Affiliations:** Division of Population Medicine, Cardiff University, Cardiff, UK; Division of Population Medicine, Cardiff University, Cardiff, UK; Division of Population Medicine, Cardiff University, Cardiff, UK; Division of Population Medicine, Cardiff University, Cardiff, UK; Primary Care and Interventions Unit, the United Kingdom Health Security Agency (UKHSA), Gloucester, UK; Primary Care and Interventions Unit, the United Kingdom Health Security Agency (UKHSA), Gloucester, UK

## Abstract

**Background:**

Urinary tract infections (UTIs) are one of the most common bacterial infections affecting women. The COVID-19 pandemic altered how healthcare was accessed and resulted in the rapid adoption of remote technologies. This study explored patients’ experiences of consultations for UTIs in general practice during the pandemic.

**Methods:**

Women included in this study were ≥16 years, recruited via Ipsos’s online panels in England, reporting at least one episode of UTI symptoms in the previous year, and had sought a consultation with a healthcare professional. We analysed 799 responses to a free-text questions, using inductive thematic analysis, regarding their experiences.

**Results:**

We identified key themes related to (i) the consultation mode and healthcare professional consulted, (ii) UTI assessment and management, (iii) validation of UTI symptoms and experience and (iv) concerns due to the COVID-19 pandemic. Positive aspects of care related to prompt and thorough assessment and treatment, consulting a healthcare professional (HCP) who validated their experience, while encouraging discussions about prevention and self-care. Negative aspects of care were related to long appointment waiting times, a lack of in-person consultation if desired and patients feeling uninformed and unvalidated about their UTIs.

**Conclusion:**

This study presents evidence that positive patient experience on UTI assessment and management is determined by the HCP involved, the mode of consultation and the application of shared decision-making to determine treatment. To improve satisfaction, systems and consultations should account for these patient preferences with shared decision-making approaches, adapted for remote consultations, to support discussions around UTI investigation and management.

## Introduction

March 2020 marked the year for unprecedented change in healthcare service delivery due to the rapid spread of SARS-CoV-2 virus and resulting severe acute respiratory syndrome coronavirus 2 (SARS-CoV-2), leading to the COVID-19 pandemic.^1^ To allow for safe healthcare access amid the lockdown and limit virus transmission, the healthcare sector, including general practice, responded with the rapid adoption of remote or virtual (e.g. telephone, video etc.), consultations.^2^ The Rapid COVID-19 intelligence to improve care response (RAPCI) project reported that 90% of consultations (88% were initially by telephone and 1% by video) with General Practitioners and 46% of nurse consultations took place remotely during April 2020.^3^ Published data from the NHS also reported a 51.5% decrease in face-to-face appointments and a 270.5% increase in telephone appointments.^4^ Research shows that remote consulting can be effective for many patients, incurring lower costs and achieving similar outcomes to face-to-face assessments.^5,6^ Although evidence shows that face-to-face consultations are still often preferred by patients, satisfaction with telemedicine consultations in primary care can be high, and is preferred in some situations.^7,8^

The incidence of several common infections declined during the pandemic, such as, non-COVID respiratory tract infections and gastrointestinal infections.^9–11^ The reasons for this are multifactorial but are likely due to the implementation of protective measures such as social isolation, mandatory use of facemasks and increased handwashing.^11,12^ The pandemic, however, had minimal effect on the incidence of urinary tract infections (UTI).^13^ UTIs are a common presentation to primary care and are more prevalent among women, with one-third experiencing one or more UTI episodes by the age of 24.^14,15^ UTIs have a significant impact on women's daily life and relationships and can provoke feelings of hopelessness and fear of recurrence.^16^ There is also the risk of serious sequelae resulting from UTIs including ascending or worsening infection causing pyelonephritis, systemic infection and sepsis, or recurrent UTIs.^14^ Treatment of UTIs is based on clinical signs, symptoms and urine sample testing in specific cases.^17^ While empirical antibiotic prescription is the first line management for confirmed UTIs, research suggests potential inappropriate or unnecessary prescribing,^18,19^ which may be exacerbated by virtual consultations as clinicians are unable to examine patients and may prescribe antibiotics to be on the ‘on the safe side’.^20,21^

Patient satisfaction is a core dimension of quality healthcare.^22^ The aim of the study was to explore patients’ experiences and satisfaction with the assessment and management of their UTIs in primary care, including interactions with healthcare, during the COVID-19 pandemic, using free-text survey responses.

## Materials and methods

### Study design and data collection

The study was run by a market research agency (Ipsos) on behalf of UK Health Security Agency (UKHSA) from 13 March to 13 April 2021. Approximately 53 000 women aged ≥16 years in England were invited through Ipsos’s online access panels. These panels consist of internet users who have agreed to take part in online market research surveys. On registration, panel members provide contact details and demographic information, and undergo validation and screening processes. Survey invitations are sent to pre-selected panellists by email including key details such as survey information, a unique access URL, Ipsos contact details, privacy policy and opt-out options. Respondents are guided through relevant questions based on their answers, and quotas are managed to ensure balanced sampling and minimize bias. Deployment is controlled via batch mail-outs and automated scheduling, with surveys closing once target quotas are met. A questionnaire that had been used to conduct a similar survey in 2014 was adapted by the research team to include additional information specific to women’s symptoms and care seeking during the COVID-19 pandemic (Supplementary material, available as Supplementary data at *JAC-AMR* Online).^18^ The e-survey collected information on participants’ baseline sociodemographic characteristics, UTI symptoms, whether they contacted a healthcare professional, the type of healthcare professional consulted, their location (e.g. general practice, accident and emergency etc), subsequent management and their experiences of the care they received during the COVID-19 pandemic. The survey also assessed consultation mode. However, because participants could choose more than one option (in person, phone, e-consult etc.), experiences related to consultation mode were based on quotes in the free-text responses.

In total, 53 000 women, who were part of the Ipsos research panel, were invited to complete the e-survey during March and April of 2021 with a retrospective recall period of March and April 2020 (during the COVID-19 pandemic).^1^ Consent was obtained through Ipsos’ web-based platform before women completing the survey.

The term ‘UTI’ was explained to potential participants at the beginning of the questionnaire as ‘Urinary Tract Infections (UTI’s) are often called urine, water or bladder infections or cystitis. They can give you pain when passing urine and a need to pass urine more often’. Women, who experienced UTI symptoms in the previous year, were asked multiple questions about the symptoms they had with the self-reported UTI, its severity and management. Women were asked about who they sought care from—this included a General Practitioner, nurse, pharmacist, healthcare assistant, A&E or NHS 111—and to score their overall satisfaction with the assessment and management they received for their most recent UTI using a 10-point Likert scale (1 to 10, 1 being dissatisfied and 10 corresponding to high satisfaction). Participants were also asked to respond, in free-text, as to why they gave the chosen satisfaction score (Box 1). Individual responses to the free-text question were extracted for this analysis.

Box 1. Satisfaction questions in e-survey questionnaire

- Did you contact a healthcare professional. (YES/NO).
- How satisfied are you with the management of your most recent UTI? (Scale of 1 to 10).
- Please tell us why you have a score of …? (Free-text answer).

### Inclusion and exclusion criteria

**Figure 1.**
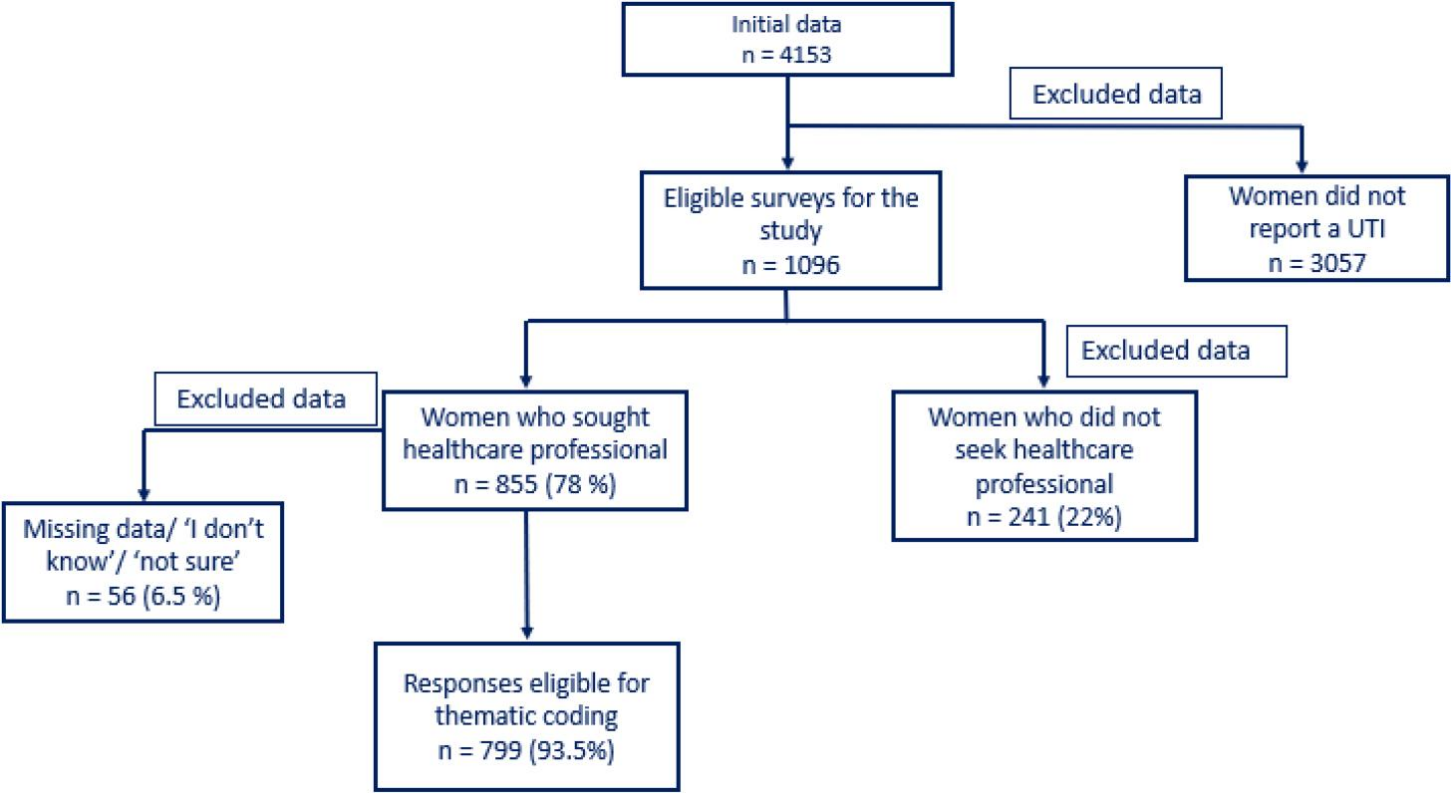
Flowchart for inclusion of data in thematic analysis.

Women included in the study were those aged 16 years old or older, reporting at least one episode of UTI symptoms in the previous year, and had sought a consultation with a healthcare professional (HCP) for the management of their UTI (Figure 1).

### Analysis

The data were initially coded by A.D. (resident doctor), and the coded data were discussed with the wider research team (L.S., H.A., E.C.). The research team included individuals with qualitative experience and expertise (L.S., E.C. and D.L.) and practising General Practitioners (L.S. and H.A.). The data were double coded by an external researcher who did not have a clinical background (L.C.).

The free-text answers from the e-survey questionnaire were thematically analysed.^23^ A.D. (resident doctor) performed inductive content analysis of the free-text answers from the e-survey using open coding, axial coding and selective coding schemes supported by NVivo (Version 12). A.D. first became familiar with the free-text answers by reading and re-reading the text. Recurring comments or patterns were noted as potential codes during the initial familiarization stage. The coding phase comprised of systematic coding of the data and determining a coding framework. A codebook was developed during the initial coding process and the codes and themes were governed by the content of the answers. The codes were developed, reviewed and amended iteratively throughout the analysis. The next step was to investigate and explore the relationship and patterns between the codes leading to the development of preliminary group of codes that subsequently became study themes. The responses were given the necessary codes to cover the content of the comment. The developed codes were discussed and reviewed with the wider research team (L.S., E.C.).

The research team included individuals with qualitative research experience, L.S. (PhD candidate and General Practitioner), E.C. (Researcher and Registered Nurse) and D.L. (Researcher and Scientist). Inconsistencies about coding were discussed with H.A. (Epidemiology Clinical Reader and General Practitioner) until a consensus was reached. This mitigated bias and ensured credibility, confirmability and allowed us to consider potential alternative explanations. The team were responsible for interpreting the analysis.

Given that the free-text responses were collected as part of a large-scale survey and were not the primary qualitative data source, data saturation was not sought. The purpose of the analysis was to capture a broad range of patient experiences to identify prevalent themes related to UTI management during the COVID-19 pandemic. The large volume (799) of responses allowed identification of consistent patterns without requiring saturation. The free-text responses were not analysed by severity of symptoms due to the potential loss of data content through categorizing the symptoms groups (severe, moderate, mild). Also, we discerned during early data analysis that there was a consistent lack of concordance between the satisfaction score and the content of the comment. Hence, satisfaction score only forms part of the descriptive results.

## Results

**Figure 2.**
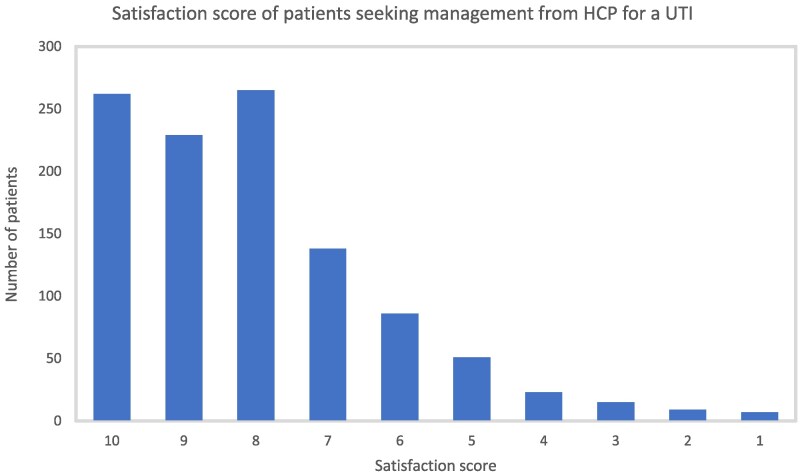
Bar chart illustrating the satisfaction level by women who sought care from HCP for management of UTI.

In total, 4153 women (∼8%, 4153/53 000) responded to the online questionnaire invitation between March and April 2021. Of them, 1096 reported a suspected UTI during the preceding year (up to March or April 2020) and 78% (855/1096) sought care from a HCP forming the study cohort. The mean age of the women in the cohort was 44.9 [standard deviation (SD) = 43.73 to 46.04]. The distribution of ethnic groups was 89.5% (765/855) of women were white, and only 4.8% (41/855), 2.4% (20/855) and 2.0% (17/855) were from Asian, black and mixed-race descent, respectively (Table 1). The majority, 51.0% (436/855), of women sought a consultation with a General Practitioner at a local surgery (Table 2). Other settings accessed included other HCPs in primary care (e.g. nurses, pharmacists in various settings etc.), out of hours or urgent care (including NHS 111), online providers and attendance at accident and emergency departments (Table 2). The majority scored their satisfaction above 8 (out of 10) (Figure 2).

**Table 1.** Baseline sociodemographic characteristics

Sociodemographic characteristic	*N*	Percentage (%)
Age		
Mean	44.9	
Standard deviation	43.73 to 46.04	
Employment		
Employed full-time	279	32.6
Employed part-time	182	21.3
Full-time parent homemaker	96	11.2
Retired	129	15.1
Self employed	40	4.7
Student/pupil	36	4.2
Unemployed and not looking for a job/long-term sick or disabled	63	7.4
Unemployed but not looking for a job	30	3.5
Total	855	100.0
Social Grade		
Upper middle class	23	2.7
Middle class	201	23.5
Lower middle class	272	31.8
Skilled working class	126	14.7
Working class	152	17.8
Lower level of subsistence	81	9.5
Total	855	100.0
Ethnic group		
White	765	89.5
Asian/British Asian	41	4.8
Black/British black	20	2.4
Mixed race	17	2.0
Other ethnic group	6	0.7
Prefer not to say	6	0.6
Total	855	100.0

**Table 2.** Descriptive statistics of the prevalence breakdown of the types of healthcare professional they sought care from

Healthcare professional	*N* ^a^	Percentage (%)
General Practitioner at local surgery		
Yes	436	51.0
Pharmacist at local surgery		
Yes	82	9.6
Out of hours GP		
Yes	40	4.7
Nurse at my local surgery		
Yes	96	11.2
Out of hours nurse		
Yes	15	1.8
Receptionist or healthcare assistant at my local GP surgery		
Yes	68	8.0
Pharmacist at my local pharmacy, chemist or supermarket		
Yes	173	20.2
Accident and Emergency		
Yes	37	4.3
Online healthcare professional on the internet provided by a pharmacy e.g. Lloyd		
Yes	36	4.2
NHS 111 by phone or online		
Yes	67	7.8
NHS walk-in or urgent care		
Yes	40	4.7

^a^Total of each healthcare service is 855.

### Identified themes

Free-text responses concerning patient’s experiences of the assessment and management of their UTIs were grouped under four thematic categories: (i) consultation mode and HCP consulted; (ii) UTI assessment and management; (iii) validation of UTI symptoms and experience and (iv) concerns due to the COVID-19 pandemic. These four major themes were highly reflected across the participant responses. Specific patient responses were reported to illustrate the main findings (Table 3).

**Table 3.** Summary of themes and subthemes

Themes	Subthemes	Example of patient quotations	Interpretation
Consultation mode and healthcare professional (HCP) consulted	HCP consultation preference	*‘Could have been more thorough or have sent me to an actual doctor.’* *‘UTI went. In my view a pharmacist is an excellent person to ask for advice as you do not have to wait for an appointment, and they are very knowledgeable.’* ‘*nurse listened carefully as I described my symptoms’.* ‘*The person I spoke to was not only sympathetic but happy to help'.*	Some women preferred seeking healthcare from a General Practitioner over an allied HCP as they perceived that they are more likely to be receive a more thorough assessment. However, some discussed how perception of receiving care from HCP changed after experiencing care for their UTI by a nurse or pharmacist. Irrespective of the type of HCP, good patient-HCP interaction reflected higher satisfaction level among patients.
	Mode of consultation	*‘Easy to use e-consult, timely reply and urine test, no need to visit surgery’.* ‘*I got everything I needed without having to leave the house’.* ‘*Never saw a face, all conducted on ‘phone, a very impersonal service.’* ‘*I wanted to see a doctor but I felt uncomfortable over the phone so I went to see the pharmacist instead’.* ‘*why cant I see a doctor wearing a mask and sitting apart … if I could see different people in the supermarket’.*	Women reported mixed feelings about virtual consultations. Some found it an efficient, effective, and a convenient approach while still maintaining safety measures. Other women were unsatisfied with online/telephone consultations as they felt it was ‘impersonal’.
	Access to healthcare	‘*Treatment and advice was good, had to wait a very long time to speak to someone.’* ‘*… I ended up paying for a private service which did both the check and the prescription same day.’* *It took to long to have treatment ‘…I was struggling to walk then 2 days later I was admitted as an emergency with sepsis.’*	The long waiting time to speak to an HCP was a significant contributor to patient dissatisfaction as their symptoms were not managed in time. Although the adequacy of the treatment was satisfactory, it was the long waiting time that negatively affected their experience. In some cases, the delay in treatment caused a complication in their UTI, progressing to sepsis. In other cases, women sought a private service for quick management of the condition.
UTI assessment and management	treatment options	*‘The antibiotics the gp prescribed worked and cleared my infection’* *I would have preferred additional information about over-the-counter treatments to prevent uti instead of using antibiotic’* ‘*The treatment was effective, but the UTI's are ongoing with no way of preventing them’*	Some women who received an antibiotic were very satisfied with the treatment as their UTI was resolved within a short period of time. Some women need more information on advice for pain relief, prevention, and self-care measures.
	Request of a urine sample	*‘Would have preferred to have had a urine test to make sure’* ‘*GP showed respect and understanding but it took two lots of antibiotics before I was given a urine test.’* ‘*Because during the pandemic on the first UTI they didn’t make me give a urine sample and gave me random antibiotics to try.’*	Treatment of UTI without a urine sample was another factor reducing patient satisfaction and affecting their confidence with choice of treatment. Patients would have preferred a urine sample test before an antibiotic was prescribed just to ‘make sure’ and not prescribe a ‘random antibiotic’. This this reduces their confidence in the effectiveness of the treatment.
Validation of UTI symptoms and experience	‘*Speedy advice confirming my own thoughts on treatment’.* ‘*No advice was given on prevention etc but the doctors trusted that I knew what it was and gave me what I needed to recover.’* ‘*i had asked for a change of antibiotic but was prescribed the same one which made the uti worse not better’* ‘*Told the UTI wasn't very prominent/severe so wasn't going to get antibiotics until I pushed for them as I knew I had symptoms and needed to get rid’* ‘*I was told Id be referred for further investigation at hospital but have heard nothing in 4 months’.*		Some women felt validated when the doctor ‘trusted’ them. Feeling reassured was another reason for women satisfaction as they derived comfort from the fact that treatment was immediate and one that matched what they expected. Lack of follow-up with test results or further investigation as well as undermining severity of symptoms made women feel invalidated and decreased their satisfaction with treatment. Some women felt invalidated because they were not prescribed an antibiotic when they needed and requested it and had to ‘push’ for an antibiotic prescription. Failing to prescribe an alternative antibiotic upon the women’s request as she was not improving was another issue women faced.
Concerns due to the COVID-19 pandemic	‘*my GP surgery has been working well and safely during COVID’* ‘*it was all very safe made a phone call to doctors surgery took my sample to the door waited for a phone* *Call at home …’* ‘*Felt covid was restriction my service from the nhs’*		Women were satisfied with the ability to access care during the COVID-19 pandemic without increasing their risk to the virus. Some women felt that the pandemic restricted their access to healthcare while other women felt that their health conditions were neglected due to the pandemic. Other women did not seek healthcare as their pain level ‘was not severe’.

### Consultation mode and healthcare professional consulted

For women who accessed a healthcare service for their UTI, most either consulted with a General Practitioner or an allied HCP (nurse or pharmacist). Patient satisfaction with the management of their UTI was driven by the nature of the interaction. Women reported higher levels of satisfaction when the experience was pleasant, such that they built rapport with the HCP and were treated in a professional and sympathetic manner.

Even though doctors, nurses and pharmacists were all praised for their competence and mannerisms, some women commented that they preferred being assessed and managed by a doctor instead of a nurse. Some women who were dissatisfied with their treatment related this to the role/expertise of the HCP they saw, commenting that their examination ‘could have been more thorough with an actual doctor’. A few women were positive about the experiences they had when seeing a nurse and compared it to be ‘exactly like a face-to-face consultation with my GP [doctor]’. The positive comments were due to the ‘friendly and professional’ nurses who were caring and attentive.

Most women understood why virtual consulting was necessary and some women were satisfied with consulting virtually. The benefits that women perceived from virtual consultations was the ability to access healthcare at home. However, some patients were dissatisfied with virtual consulting stating that discussing their UTI symptoms over the phone was ‘embarrassing’ and ‘uncomfortable’ or choosing to attend to their local pharmacy instead of speaking to a General Practitioner over the phone. For some women, virtual consultations acted as a barrier making it ‘a bit hard’ to consult a doctor and get antibiotics.

### UTI assessment and management

Many women, who reported higher satisfaction, stated this was because they were offered prompt antibiotic treatment which resolved their symptoms quickly. Antibiotic prescription is perhaps correlated with a satisfied patient if their UTI symptoms resolved in a short period of time causing ‘no disruption’ to their daily activities. Some women were also satisfied with being given antibiotic alternative treatments thus avoiding antibiotics and its associated side-effects.

A conservative approach, prescribing a delayed antibiotic, advising increased fluid intake, and use of over-the-counter analgesics, was well received by some women. Women were satisfied with being recommended ‘cranberry juice’ or ‘cystitis sachets’ as it helped relieve the symptoms but also ‘avoid having to go to the doctors for ‘antibiotics’. Some women were satisfied with advice about ‘what they could take and what to do’ and safety netting advice if the UTI progresses. However, many women, especially those who suffer from recurrent UTIs, complained about the lack of advice they have received about how to prevent UTI recurrence.

An antibiotic prescription, without performing a urine dipstick had a negative impact on the trust and confidence the women had in the treatment decision. Some women alluded to the fact that they were still prescribed an antibiotic ‘without further testing’ even though the urine test ‘showed no UTI’. A recurring reason why some women were dissatisfied with the treatment was due to antibiotics being prescribed without information from a urine dipstick and being prescribed ‘random antibiotics to try’. This highlights that women value urine sample testing as it reassures them that treatment is offered on grounds of sufficient clinical evidence. Another reason for women’s dissatisfaction was the long wait before being able to access healthcare, irrespective of type of HCP. Some women’s symptoms worsened, and they attributed this to the delayed assessment and treatment. A few patients experienced significant pain and could not wait, eventually accessing private healthcare; this caused distress and incurred a cost.

### Validation of UTI symptoms and experience

Women satisfied with treatment felt understood, reassured and validated. Women who suffer with frequent UTIs have an ‘experience with UTI’ therefore ‘know that antibiotics would clear it up’ and the doctor would understand this and provide a prescription promptly. One woman explicitly said that the doctor ‘trusted’ her judgement and prescribed her antibiotics for her symptoms accordingly. Other women who have co-morbidities such as diabetes, heart problems or are immunocompromised reported that their underlying conditions were taken into consideration and management was tailored to their needs. This reflected a positive outcome as they derived comfort from the fact that healthcare staff listened, trusted and understood their needs.

Some women with experience of UTIs, said they may clash with the doctor’s decision regarding the type of treatment they receive. A few women, who were dissatisfied with their management, claimed that the General Practitioner was dismissive of their symptoms and ‘said there was not any issue, so I had to contact NHS 111’. Some women expressed dissatisfaction despite receiving treatment as they felt that consultation with the doctor was ‘a little rushed’. A few women were dissatisfied as they requested a change of antibiotic but were prescribed the same one which they reported caused their symptoms to persist. A few women regarded the conservative approach to managing a UTI, one that does not include an antibiotic prescription, as not ‘being offered real help’. The prominent response correlating dissatisfaction was when women were not prescribed an antibiotic. Some women had to ‘push’ for an antibiotic prescription while other women felt that since they could not get a prescription, they felt that they were not being listened to and therefore ‘wasted own time and the doctors time’.

### Concerns during COVID-19 pandemic

Free-text responses about the COVID-19 pandemic specifically were limited. However, given the predicament that altered how the healthcare system functioned a lot of women were very appreciative of how their issues were ‘dealt with efficiently’. Women understood why measures were put in place, and they expressed satisfaction as they received care in a safe environment without increasing their risk of contracting COVID-19. Some women were extremely dissatisfied, and they felt that other health conditions were not prioritized due to the pandemic. Some women also commented on how the COVID-19 pandemic ‘restricted’ them from seeking help from the NHS.

## Discussion

This study explored women’s satisfaction with the assessment and management of their self-reported UTIs during the COVID-19 pandemic using free-text responses correlating with management satisfaction. It highlighted aspects of patient experience with assessment and management that influence satisfaction level including type of HCP offering the assessment treatment, the mode of consultation (face-to-face or remotely), the prescription of antibiotics, discussing alternative treatment and urine testing. Findings showed that satisfaction was often linked to the consultation and management process, especially patient belief around the HCP’s ability to listen to and validate their symptoms, experience and concerns. Comments also highlighted that a long waiting time for appointments delayed appropriate assessment and treatment, which worsened UTI symptoms.

### Comparison with existing literature

A systematic review by Sanyaolu *et al.* exploring the experiences and views of women with recurrent UTIs reported that they commonly used self-help and lifestyle changes to try to prevent and avoid UTIs.^24^ Providing self-management and lifestyle advice also ‘empowered women by providing some control over their UTIs’.^24^ They also reported that some women actively used complementary and alternative medicine, such as herbal medicines, as alternatives to antibiotics.^24^ Studies have also identified that women experiencing UTI symptoms were open to an alternative antibiotic therapy, some even preferred this approach.^18^ This aligns with the findings in our study as women, especially those with recurrent UTIs, expressed their disagreement with the continuous antibiotic use and were interested in understanding measures they can employ to prevent them recurring. Interestingly, some women in our study described being satisfied with a delayed prescription, in keeping with UK guidance in certain scenarios, or being recommended cranberry juice as an alternative to antibiotics.^25^ This is in keeping with previous research that demonstrates that women with UTIs commonly use this approach.^18,26^ However, despite this preference to use cranberry-based products, evidence for acute or preventive management is limited and inconclusive, with current UK guidelines also acknowledging this uncertainty about effectiveness.^25,27–29^

A qualitative study investigating women’s understanding around urine testing for the assessment of a UTI demonstrated that some women regarded a urine test as a necessary tool to aid treatment decisions particularly for the prescription of the ‘right antibiotic’.^30^ While some women were conflicted about how the information from a urine sample test guided management, other women felt relieved that their urine sample is checked rather than ‘just being given antibiotics’.^30^ Findings in our study consistently reported that women expected a urine sample to be tested before being prescribed antibiotics as it made them feel reassured that the correct treatment is provided. This is in contrast with the systematic review by Sanyaolu *et al*, where some women with recurrent UTIs felt that acute investigation for a UTI was unnecessary as ‘the diagnosis is obvious’.^24^ This is further supported by a Dutch study highlighting that a urine test can be ‘unnecessary’ as the patient was certain that they have a UTI, and they felt that antibiotics will not be prescribed if a urine test was not done.^31^ The same study discussed urine testing as means of aiding diagnosis and optimizing prescription of antibiotics. Similar to other studies our findings demonstrate that, irrespective of their experience with UTIs, some patients lack knowledge about why a urine test is done. Current UK guidance advises the use of urine dipstick in selected cases to aid diagnosis and this emphasizes the importance of a detailed explanation about urine testing so that patients are aware when urine test is recommended.^31,32^

Comments from women indicated they were more satisfied with treatment if they felt understood, reassured and validated. This aligns with the finding from studies by Leydon *et al.* and Lecky *et al.* where both concluded that a key reason why women reported a positive experience was feeling that their symptoms and their views were validated.^33,34^ In addition, a qualitative study by Cox *et al.* found that patients who felt like they were ‘taken seriously’ and involved in the process of shared decision-making for their treatment were more satisfied with the consultation, also finding that women with more experience with UTI had different expectations from the consultation than women who had little or no experience.^35^ This highlights the need for protected time and resources to discuss UTI management with women, according to their experience, so they are able to contribute to shared decision-making around management, especially when consultations are virtual.

Most women in the sample accessed healthcare (excluding community pharmacy) by phone (67%), with fewer attending in-person appointments (16%) or using other online services.^36^ Feedback on virtual versus in-person consultations in this study was mixed: some appreciated the convenience of remote appointments, while others preferred the personal connection and privacy of face-to-face care. Research in England also highlighted the importance of personal preference on the mode of consultation, finding that patients who requested in-person consultations for UTI but were given a phone consultation reported lower patient-rated confidence and trust in health professionals.^28^ A rapid review exploring both patient and HCPs experiences of remote consulting in several countries during the COVID-19 pandemic demonstrated similar findings.^37^ Patients described the convenience of remote consulting and how it reduced this risk of COVID-19 transmission but also reported several disadvantages including that consultations felt ‘impersonal’ and created additional communication barriers. As studies on managing UTIs remotely versus in person show variable influence on management outcomes in different groups, it is important to develop or tailor tools and resources to support management, communication and shared decision-making through remote consultations.^38–40^

### Strengthens and limitations

An important strength of this study is the large data sample included in the final thematic analysis allowing for a broad representation of women from different social classes in England. The use of an online survey may have encouraged women to be more open about their UTI symptoms and their experience with the care they received compared with data collected from an interviewer. However, a limitation in this context is that an online survey excluded women who are less technologically affluent, who do not have access to the internet or unable to access the e-survey due to a disability; this may lead to selection bias towards certain participant groups.

Another limitation of this study is that there was no follow-up or further communication with participants who responded to the free-text questions; therefore, we did not have the opportunity to explore other things that might have affected care, e.g. urine testing, ease of care access, explore how findings may have been connected or the opportunity to clarify the context or meaning of the responses. Some participants commented on the mode of consultation, but we did not have this information for every participant as the questionnaire allowed multiple modalities to be selected. Therefore, it was unclear which specific modality the participant was referring to unless it was explicitly stated. This meant we could not describe or explore how consultation mode might have contributed to the different experiences between HCPs consulted, experience of management or validation of UTI symptoms fully. An important limitation was the lack of concordance between the Likert scale satisfaction score and the content of the free-text responses. Some participants reported high scores despite expressing negative comments, and vice versa, limiting the validity of the quantitative data in capturing the nuances of patient experiences. As a result, the satisfaction scores were excluded from the analysis, and we were unable to stratify care-seeking data with satisfaction outcomes. A further potential limitation is retrospective recall, which could have been up to 12-months, thus it is possible that recall bias can affect the patient’s memory of their experience regarding how their UTI was managed and the reported experience. Most of participating women (89.5%) were white British, therefore responses may have been different if individuals from black, Asian and ethnic groups were more represented in the study. Owing to the lack of representation by individuals from black, Asian and ethnic minority groups, this may suggest that findings from this study are not transferrable to the wider general practice population.

### Conclusion

In summary, this study highlighted that women were mostly satisfied with the assessment and management of their UTI if they received thorough assessment, prompt administration of antibiotics, and were treated in a sympathetic and professional manner. It also revealed that some women would prefer to discuss alternative options before being prescribed an antibiotic. Finally, further development of available patient decision-making tools and resources, that can be adapted for use in virtual consultations, will help address knowledge gaps on UTIs.

## Supplementary Material

dlaf241_Supplementary_Data
