# Supplementary material for: Women’s experiences of the assessment and management of urinary tract infections during the COVID-19 pandemic: a qualitative analysis of free-text comments from a national survey in England
Source: JAC Antimicrob Resist. 2025 Dec 19;7(6):dlaf241. doi: 10.1093/jacamr/dlaf241 (PMC12715501; doi:10.1093/jacamr/dlaf241)
Supplement: dlaf241_Supplementary_Data [file dlaf241_supplementary_data.docx]

**Supplementary material**

This document contains the supplementary material for the paper: **Patient experiences of the assessment and management of urinary tract infections during the COVID-19 pandemic: A qualitative analysis of free-text comments from a national survey in England**.

Below is the questionnaire participants were asked to complete. Response positions in the questionnaire were either fixed or randomized between questionnaires, as highlighted below. Whether participants could answer questions with a single, multiple or open-ended response is also highlighted and latter questions are based on earlier responses to the questionnaire.

Questionnaire

The next set of questions is about what you do when you have a possible urine / water infection or cystitis (commonly known as UTI) and the medicines which are prescribed for treating these infections.

We want to find out how you take care of these infections.

**ASK ALL WOMEN**

**QX01**Urinary Tract Infections (UTI’s) are often called urine, water or bladder infections or cystitis. They can give you pain when passing urine and a need to pass urine more often.

Have you EVER had a UTI?

**SINGLE CODE**

1. Yes
2. No
3. Don’t Know **FIX**

**ASK ALL WHO CODE YES TO QX01**

**QX02** Approximately how many UTIs have you had in the last year (since March 2020). Please include instances where you have had more than one course of treatment to treat the same UTI as one instance.

**SINGLE CODE**

1. 1
2. 2
3. 3-4
4. 5 or more
5. I have not had a UTI in the last year **FIX**
6. Don’t Know **FIX**

**ASK ALL CODING 1-5 or more AT QX02**

**QX02A** Approximately when was your last UTI?

**SINGLE CODE**

1. In the last 3 months (since December 2020)
2. 3-6 months ago (between September and November 2020)
3. 6 months to 9 months ago (June to August 2020)
4. 9 to 12 months ago (March to May 2020)
5. Don’t know **FIX**

**ASK ALL CODING 1-5 OR MORE AT QX02**

**QX03** Thinking about your most recent UTI, how severe, if at all, were your symptoms? Please give your answer on a scale of 0 to 10 where 0 means you had no symptoms at all and 10 means your symptoms were extremely severe.

**SINGLE CODE, FORWARD AND REVERSE SCALE**

0 - I had no symptoms at all

1

2

3

4

5

6

7

8

9

10 – Symptoms were extremely severe

11. Don’t know **FIX**

**ASK ALL CODING 1-5 OR MORE AT QX02**

**QX03a** To what extent, if at all, did your most recent UTI affect your daily life?

**SINGLE CODE, FORWARD AND REVERSE SCALE**

1. It affected it a great deal
2. It affected it a fair amount
3. It didn’t affect it very much
4. It didn’t affect it at all
5. Don’t know **FIX**

**ASK ALL CODING 1-5 OR MORE AT QX02**

**QX03aa** Thinking about your most recent UTI, did you have any of the following signs or symptoms?

Select all that apply.

**MULTI PUNCH1-15, RANDOMISE 1-14**

1. I had to pass urine more often in the night than usual
2. I could see that my urine was cloudy
3. I had burning pain when passing urine
4. I had to pass urine more often than usual
5. I could see blood in my urine, which was not due to a period
6. I had new or worse incontinence (wetting yourself more often than usual)
7. I had pain in my lower tummy
8. I had vaginal discharge
9. I was more confused than usual
10. I was more unsteady on my feet than usual
11. I had a lower temperature than usual
12. I had a fever
13. I had rigors/shivering/shaking
14. I had kidney pain or tenderness in my back under my ribs
15. Other – please list **FIX**
16. I can’t remember **SINGLE CODE FIX**
17. None of these **SINGLE CODE FIX**

**ASK ALL CODING 11 OR 12 AT QX03AA (FEVER OR RIGORS/SHIVERING/SHAKING)**

**QX08B** Did you have a COVID test because of your symptoms with your most recent UTI?

**SINGLE CODE**

1. Yes
2. No
3. I can’t remember

**ASK ALL CODING YES AT QX08B**

**QX08C** Was this COVID test positive?

**SINGLE CODE**

1. Yes
2. No
3. I can’t remember

**ASK ALL CODING 1-5 OR MORE AT QX02**

**QX05** Which, if any, of the following healthcare professionals did you contact as a result of your most recent UTI? This could be could be in person, on the telephone, via email or other means.

Select all that apply

**MULTI CODE 1-12, RANDOMISE 1-11, KEEP CODES 1-4 TOGETHER**

1. GP at my local GP surgery
2. Nurse at my local GP surgery
3. Pharmacist at my local GP surgery
4. Receptionist or other health care assistant at my local GP surgery
5. Out of hours GP
6. Out of hours Nurse
7. Pharmacist at my local pharmacy, chemist or supermarket
8. Online healthcare professional on the internet provided by a pharmacy e.g. Lloyds or Superdrug
9. A&E, (Accident and Emergency)
10. NHS 111 by phone or online
11. NHS walk–in or urgent care centre
12. Other (please specify) **FIX**
13. None - I didn’t contact a healthcare professional **SINGLE CODE FIX**
14. Don’t know **SINGLE CODE FIX**

**NEW SCREEN**

The next few questions focus on what happened when or after you contacted the healthcare professional.

**ASK ALL NOT CODING 7 (PHARMACIST AT MY LOCAL PHARMACY, CHEMIST OR SUPERMARKET) OR 13 (NONE), AT QX05)**

**QX06** How did you contact the health care professional(s)?

Remember you may have contacted more than one healthcare professional and consulted in more than one way

Select all that apply

**MULTI CODE 1-7, RANDOMISE 1-6**

1. I spoke to them over the phone
2. I spoke to them on a video call
3. I spoke to them face to face in person
4. I spoke to them on-line without a video (internet call)
5. I consulted on the internet without speaking to them (for example web chat)
6. I completed a form and returned it – for example e-consult or My GP APP
7. I consulted them by SMS or text message
8. Other
9. Don’t know **SINGLE CODE FIX**

**ASK ALL CODING 7 AT QX05 (PHARMACIST AT MY LOCAL PHARMACY, CHEMIST OR SUPERMARKET)**

**QX10** How did you contact the pharmacist at your local pharmacy, chemist or supermarket? Remember you may have contacted more than one healthcare professional there and consulted in more than one way.

Select all that apply

**MULTICODE 1-7, RANDOMISE 1-6**

1. I spoke to them over the phone
2. I spoke to them on a video call
3. I spoke to them face to face in person
4. I spoke to them on-line without a video (internet call)
5. I consulted on the internet without speaking to them (for example web chat)
6. I completed a form and returned it – for example e-consult
7. In another way, please specify **FIX**
8. Don’t know **SINGLE CODE FIX**

**ASK ALL CODING 7 AT QX05**

**QX10A**

Thinking of your most recent UTI, what advice or treatment, if any, were you given by the pharmacist when you contacted them at your local pharmacy, chemist or supermarket? Select all that apply

**CODES 1-11 ARE MULTICODE, RANDOMISE CODES 1-10**

1. I was given advice about my symptoms
2. I was advised about treatment I could get without a prescription
3. I was advised about pain relief (such as Paracetamol, or Ibuprofen)
4. I was given a UTI leaflet or directed to more information about UTIs
5. I was advised where to seek further help if my symptoms got worse or did not improve
6. I was given advice about how to prevent a future UTI

I was advised to drink more fluids

1. I was referred to a COVID clinic to discuss my symptoms
2. I was advised to contact a GP, NHS walk-in or urgent care centre/A&E
3. I was advised to contact another healthcare professional
4. Other (please specify) **FIX**
5. I wasn’t given any advice or other treatment **SINGLE CODE FIX**
6. Don’t know **SINGLE CODE FIX**

**ASK ALL CODING 1-5 OR MORE AT QX02**

**QX06A** Thinking about your most recent UTI, did you provide a urine sample for testing?

**SINGLE CODE**

1. Yes, I did provide a urine sample
2. No, I did not provide a urine sample
3. Don’t know
4. Prefer not to say

**ASK THOSE CODING 1 @ QX06A**

**QX06AB** Who requested the urine sample?

**CODES 1, 2, 4 ARE MULTICODE**

1. A urine sample was requested by a pharmacist in a pharmacy, chemist, or supermarket
2. A urine sample was requested by my GP surgery, out of hours, walk-in centre, clinic, or other healthcare provider
3. A sample was not requested, but I provided a urine sample anyway to the GP surgery/ out of hours/walk-in centre, etc
4. Other (please specify)
5. Don’t know **SINGLE CODE**

**ASK ALL CODING 1-3 AT QX06AB**

**QX06AA** What did the results of the urine sample show from your most recent UTI?

**SINGLE CODE, RANDOMISE 1-3**

1. I received the result and was told I had a UTI
2. I received the result and was told I did not have a UTI
3. I received the result and was told I needed a different antibiotic
4. I did not receive any results
5. Other, please specify **FIX**
6. I don’t remember **FIX**

**ASK ALL CODING 1-5 OR MORE AT QX02**

**QX06B**

Still thinking about your most recent UTI, were you prescribed antibiotics?

**SINGLE CODE**

1. Yes, I was prescribed antibiotics

2. No, I was not prescribed antibiotics

**ASK ALL CODING 1 at QX06B**

**QX06BA**

Who prescribed the antibiotics?

**SINGLE CODE**

1. A healthcare worker (GP, nurse or pharmacist) at my GP surgery
2. A pharmacist in my local pharmacy, chemist or supermarket (this does not include collecting a prescription from a pharmacy)
3. A healthcare professional at a walk-in centre, clinic or out of hours centre
4. Other
5. Don’t know

**ASK ALL CODING 1 AT QX06B**

**QX06BB**

How were the antibiotics prescribed to you?

**SINGLE CODE**

1. I was prescribed antibiotics over the phone
2. I was prescribed antibiotics over the internet
3. I was prescribed antibiotics in person during a face to face consultation
4. Other
5. Don’t know

**ASK ALL CODING 1 at QX06B**

**QX06BC** What type of prescription were you given?

**SINGLE CODE**

1. I was prescribed antibiotics to take as soon as possible
2. I was prescribed delayed/ back-up antibiotics (this is a prescription you were given at the time of diagnosis, but you are advised only to take them if your symptoms did not improve or got worse after several days)
3. I was prescribed standby antibiotics (this is when you are prescribed antibiotics at the time of diagnosis, but only use if the infection occurs again in the future)
4. Don’t know

**ASK ALL CODING 1-12 at QX05**

**QX06C** Thinking of your most recent UTI, what **<IF CODED 1-7 AT QX10A INCLUDE THE WORD “**other**”>** advice or **<IF CODED 1 AT QX06B INCLUDE THE WORD “**other**”>** treatment, if any, were you given? Select all that apply

**CODES 1- 11 ARE MULTI PUNCH, RANDOMISE CODES 1-9 KEEP 9-10 TOGETHER**

1. I was prescribed treatment other than antibiotics for my symptoms

2. I was given advice about my symptoms

3. I was advised about treatment I could get without a prescription

4. I was advised about pain relief (such as Paracetamol, or Ibuprofen)

5. I was given a UTI leaflet or directed to more information about UTIs

6. I was advised where to seek further help if my symptoms got worse or did not improve

7. I was given advice about how to prevent a future UTI

8. I was advised to drink more fluids

9. I was advised to get a COVID test

10. I was referred to a COVID clinic to discuss my symptoms

11. Other (please specify) **FIX**

12. I wasn’t given any advice or other treatment **SINGLE CODE FIX**

13. Don’t know **SINGLE CODE FIX**

**IF QX06C=CODES 1-8, THEN ASK VQ06D**

**QX06D** Where did you get this information or advice on your treatment or symptoms for your most recent UTI? Select all that apply

**multiCODE 1-8, RandomISE order 1-7**

1. the hospital
2. the GP surgery
3. the pharmacy
4. the walk-in centre / minor urgent care centre / minor injuries centre
5. a GP out of hours service
6. a hospital accident and emergency (A&E)
7. When I contacted NHS 111 or other health line
8. Other (please specify) **FIX**
9. Don’t know **SINGLE CODE FIX**

**ASK ALL CODING 1-5 OR MORE AT QX02**

**QX04**

And which of the following actions, if any, did you take as a result of your most recent UTI?

**MULTI CODE, ALLOW DK AND NULL, KEEP 1 AND 12 TOGETHER RANDOMISE CODES ANCHOR 13 and 14)**

1. I used information about UTI from NHS choices
2. I took pain relief (such as Paracetamol, or Ibuprofen)
3. I took cranberry juice or cranberry tablets or capsules
4. I took extra fluids, other than cranberry juice
5. I took the prescribed antibiotics **<Show only if code 1 “**prescribed antibiotics**” selected at QX06BC>**
6. I took the delayed antibiotic (This is when a prescription is written at the time of diagnosis, but is only taken if you feel no better or feel worse after several days) **<Show only if code 2 selected at QX06BC>**
7. I took the standby antibiotic I had at home which was prescribed for me by my GP or nurse if the infection occurred again in the future **<Show only if code 3 selected at QX06BC>**
8. I took left-over antibiotics I had at home which were prescribed for a previous illness
9. I took cystitis sachets
10. I used information about UTI given to me, or directed to, by a health professional
11. I took antibiotics I obtained without a prescription
12. I used information about UTI from the internet which was not from NHS choices
13. Other (please specify) **FIX**
14. I did not take any actions for my most recent UTI **SINGLE CODE FIX**
15. Don’t know **SINGLE CODE FIX**

**ASK ALL CODING 1 AT QX06B AND 5, 6, OR 7 AT QX04**

**QX06BD** You said you were prescribed and took antibiotics for your most recent UTI. Did you complete the course of antibiotics?

**SINGLE CODE**

1. Yes, I completed the full course of antibiotics
2. No, I did not complete the full course of antibiotics
3. Don’t know

**SCRIPTING: QUESTION TEXT INSERTS FOR QX07**

1. **IF (7 OR 8 OR 11 AT QX04) AND (NONE (13) OR DK (14) AT QX05) INSERT:**

**<**‘You said you **<IF CODE 7 at QX04 INSERT ‘**took stand-by antibiotics**’, IF CODE 8 at QX04 INSERT ‘**took left-over antibiotics you had at home**’, IF CODE 11 at QX04 INSERT ‘**took antibiotics obtained without a prescription**’>** but did not contact a health professional. Why did you treat your most recent UTI in this way?**’>**

1. **IF (7 OR 8 OR 11 NOT CODED AT QX04) AND (3 AT QX06) AND (1-6,8-12 AT QX05) INSERT:**

**<**‘You said you spoke to a healthcare professional **in person** with your most recent UTI. Why did you do this?**’>**

1. **IF (7 OR 8 OR 11 NOT CODED AT QX04) AND (1, 2,4,5,6,7 AT QX06) AND (1-12 AT QX05) INSERT:**

**<**‘You said you contacted a healthcare professional with your most recent UTI. Why did you do this?**’>**

1. **IF (7 OR 8 OR 11 CODED AT QX04) AND (3 AT QX06) AND (1-6,8-12 AT QX05) INSERT:**

**<**‘You said you spoke to a healthcare professional in person with your most recent UTI AND you took antibiotics **you** had at home. Why did you treat your most recent UTI in this way?**’>**

1. **IF** **(7 OR 8 OR 11 CODED AT QX04) AND (1, 2,4,5,6,7 AT QX06) AND (1-12 AT QX05) INSERT:**

**<**‘You said you contacted a healthcare professional with your most recent UTI AND you took antibiotics you had at home. Why did you treat your most recent UTI in this way?**’>**

1. **IF (CODE 7 AT QX05) AND (3 AT QX06)** **INSERT:**

**<**‘You said you spoke to a pharmacist in-person at your local pharmacy, chemist or supermarket with your most recent UTI? Why did you do this?**’>**

1. **IF (13 at QX05) AND NOT (ANY 7 OR 8 OR 11 at QX04)** **INSERT:**

**<**‘You said you did not contact a healthcare professional for your most recent UTI. Why was this?**’>**

**ASK ALL CODING 7 OR 8 OR 11 AT QX04 OR 1-13 AT QX05**

**QX07**

**<INSERT QUESTION TEXT>**

**MULTICODE 1-23, RANDOMISE 1-22**

1. The symptoms were severe
2. After several days the symptoms hadn’t improved
3. My friend or family suggested it
4. Because I already have another medical condition
5. I wanted an antibiotic prescription **DO NOT SHOW IF TEXT INSERT 1 OR 7**
6. To arrange a urine test **DO NOT SHOW IF TEXT INSERT 1 OR 7**
7. I needed a sick/fit note **DO NOT SHOW IF TEXT INSERT 1 OR 7**
8. I usually seek advice with these symptoms **DO NOT SHOW IF TEXT INSERT 1 OR 7**
9. I couldn’t get an appointment at my GP practice **(DO NOT SHOW IF (TEXT INSERT 2, 4, 7) OR (1 or 2 or 11 CODED AT QX05)**
10. I couldn’t wait until the GP surgery was open **(DO NOT SHOW IF (TEXT INSERT 2, 4, 7) OR (1 or 2 or 11 CODED AT QX05)**
11. Because I have recurrent UTI
12. I was worried about catching COVID **DO NOT SHOW IF TEXT INSERT 2, 4 OR 6**
13. I didn’t want to take up NHS staff time **DO NOT SHOW IF TEXT INSERT 2 OR 4**
14. I didn't want to risk spreading COVID to anyone **DO NOT SHOW IF TEXT INSERT 2, 4 OR 6**
15. There was no face to face consultation offered **DO NOT SHOW IF TEXT INSERT 2 OR 4**
16. A health professional advised that I did not need to be seen in person **DO NOT SHOW IF TEXT INSERT 1, 2 OR 4**
17. I was self-isolating **DO NOT SHOW IF TEXT INSERT 2, 4 OR 6**
18. The symptoms were not severe enough **DO NOT SHOW IF TEXT INSERT 2 OR 4**
19. I had antibiotics at home
20. I wanted treatment as quickly as possible
21. Because I did not want to take antibiotics **<ONLY SHOW IF (NOT CODING 1 AT QX06B) AND (NOT CODING 5-8 OR 11 AT QX04)>**
22. I was asked to attend in-person for a consultation **SHOW FOR TEXT INSERT 2 OR 4 ONLY**
23. Other (please specify) **FIX**
24. I don’t remember **SINGLE CODE FIX**

**ASK ALL CODING 1-5 OR MORE AT QX02**

**QX09**  How satisfied are you with were you with the management of your most recent UTI? Please give your answer on a scale of one to ten where one means you are not at all satisfied and ten means you are very satisfied.

**SINGLE CODE, FORWARD AND REVERSE SCALE 1-10**

1. 1 = not at all satisfied
2. 2
3. 3
4. 4
5. 5
6. 6
7. 7
8. 8
9. 9
10. 10 = very satisfied
11. Don’t know **FIX**

**ASK ALL CODING 1-5 OR MORE AT QX02**

**QX10** Please tell us why you gave a score of **<INSERT SCORE FROM QX09, IF 1 OR 10 GIVEN, DO NOT INCLUDE TEXT>?**

**OPEN ENDED**
